# Physiological and Biochemical Responses Induced by Plum Pox Virus and Plum Bark Necrosis Steam Pitting Associated Virus in Tuscany Autochthonous Plum cv. Coscia di Monaca

**DOI:** 10.3390/plants12183264

**Published:** 2023-09-14

**Authors:** Athos Pedrelli, Gian Piero Ricci, Alessandra Panattoni, Cristina Nali, Lorenzo Cotrozzi

**Affiliations:** Department of Agriculture, Food and Environment, University of Pisa, Via del Borghetto 80, 56124 Pisa, Italy; athos.pedrelli@phd.unipi.it (A.P.); g.ricci31@studenti.unipi.it (G.P.R.); alessandra.panattoni@unipi.it (A.P.); lorenzo.cotrozzi@unipi.it (L.C.)

**Keywords:** leaf pigments, organic acids, photosynthesis, plum bark necrosis steam pitting disease, *Prunus*, regulated non-quarantine pests, sharka disease, sugars, water status

## Abstract

The present study focused on trees of Tuscany autochthonous plum cv. Coscia di Monaca in order to evaluate the presence of viruses and elucidate the physiological and biochemical responses to virus infections under real field conditions. Among the several investigated viruses, plums tested positive only to plum pox virus (PPV) and plum bark necrosis steam pitting associated virus (PBNSPaV), occurring as both singular and co-infections. This is the first report of PBNSPaV in a Tuscany orchard. Furthermore, the present study not only confirmed the detrimental effects of PPV on the carbon dioxide assimilation rate due to both stomatal limitations and mesophyll impairments, but also showed that although PBNSPaV did not induce such photosynthetic impairments when occurring as singular infection, it enhanced this damaging effect when present as a co-infection with PPV, as confirmed by a severe decrease in the chlorophyll content. Infection-specific responses in terms of accessory pigments (i.e., carotenoids and xanthophylls), as well as sugars and organic acids, were also reported, these being likely related to photoprotective mechanisms and osmotic regulations under virus-induced oxidative stress. Overall, the results here presented represent an important step to fill knowledge gaps about the interaction of plant viruses and autochthonous *Prunus* cultivars.

## 1. Introduction

Plums are a group of stone fruits with an edible fleshy mesocarp produced by different species of the large and worldwide-distributed genus *Prunus* (family Rosaceae), mostly used by the food-processing industries and readily eaten fresh by consumers. The most important cultivars belong to *P. domestica* and *P. salicina* (European and Japanese plum, respectively [1]). Since Ancient Rome, the Mediterranean climate and the varied soils and farming conditions favoured the diffusion of European plum (native to Asia) in Italy, where this species experienced a considerable diversification. However, according to the global market, the rich Italian germplasm (estimated in hundreds of varieties) was gradually replaced by a few improved Japanese plum cultivars (mainly of American origin) because of their appreciated pomological features and adaptability to early production and post-harvest [2]. Today, Italy plum cultivation, which is in the top ten at the world level, is indeed mostly represented (ca. 75%) by Japanese plums [2,3,4]. However, some Italian farmers are still maintaining the cultivation of traditional European cultivars, mostly for local fresh markets and processing, as they are gaining growing attention because of a high perceived value and reputation deriving from their geographical origin and enhanced flavour and taste, as well as their adaptability to low-input agriculture, traditional farming, and local environmental conditions, although their yields have not always been fully satisfactory [2,5].

Given the worldwide importance of plums, viruses affecting these species represent a major economic problem due to their wide distribution and the fruit-yield losses and/or quality deterioration caused in the various host species [6]. All *Prunus* viruses are easily transmitted by vegetative propagation techniques (e.g., grafting), while some of them are also spread by vectors, such as insects, mites, and nematodes [7,8]. The control of plum viral diseases is principally based on prophylactic measures including certification programs, the confinement of suspicious material in quarantine, and the control of vectors, since eradication of infected plants is the only action when previous measures have failed, as the recovery of virus-infected material can be achieved only using expensive and time-consuming lab processes such as thermotherapy [9]. As a consequence, the European Union has introduced a specific regulation concerning plum propagating materials and classifying most plum viruses as ‘regulated non-quarantine pests’ (RNQP) in order to control their spread and reduce their negative impacts [10], although they are already widespread in Europe and endemic in some areas, including Italy [11,12].

Among the RNQP viruses challenging plums, the most important and detrimental one is plum pox virus (PPV; family Potyviridae; genus *Potyvirus*), the etiological agent of sharka disease (also known as ‘plum pox’), which caused economic losses estimated in about EUR 10,000 million in the last 30 years [13,14,15]. The main PPV strains are Dideron (PPV-D), Marcus (PPV-M), and Recombinant (PPV-Rec), with PPV-M being recognized as the most aggressive, efficiently transmitted by aphids and usually associated with severe disease symptoms [16]. However, PPV infection has been commonly associated with other RNQP viruses in *Prunus* species (e.g., apple chlorotic leaf spot virus, ACLSV; apple mosaic virus, ApMV; prune dwarf virus, PDV; myrobalan latent ringpot virus, MLRSV; prunus necrotic ringspot virus, PNRSV; [17]). In 2021, the plum bark necrosis steam pitting associated virus (PBNSPaV; family Closteroviridae; genus *Ampelovirus*), the etiological agent of plum bark necrosis steam pitting disease, which has a worldwide distribution [18,19], was reported for the first time in Tuscany, i.e., central Italy [20]. 

Hosts and plant viruses interact profoundly and modulate the dynamics and genetic structure of each other [21]. Although PPV is one of the most-studied plant viruses, and there have been major advances in detection techniques, genome characterization and organization, gene expression and transmission, and the description of candidate genes involved in PPV resistance, information concerning the plant responses to PPV infection is very scarce [22]. Overall, most of the changes produced by PPV infection have been shown to be related to photosynthesis, antioxidant defense responses, and the primary and secondary metabolisms, but many features could influence these results, such as the *Prunus* species or cultivar, PPV strain, presence or absence of symptoms, type of tissue, and duration of infection [23]. A further aspect that would probably increase such divergences is represented by co-infection with other plum RNQP, which commonly occur in the field [12], but which have never been investigated from this point of view (to the best of our knowledge), even when occurring individually. Furthermore, no study has focused on viruses’ occurrence and effects in Italian autochthonous plums, although some studies have investigated the chemical, qualitative, and nutraceutical properties of these cultivars [24,25], confirming the research interest in this plant material.

To address these knowledge gaps, autochthonous plum trees cv. Coscia di Monaca grown in the Arezzo district (Tuscany, central Italy) were selected to (1) evaluate single or multiple infections by plum viruses classified as RNQP by the European Union [10] and (2) elucidate the physiochemical responses of plum trees challenged by these RNQP. We anticipate that the outcomes reported here will be useful for the development of new strategies to cope with these diseases.

## 2. Results

### 2.1. Virus Detection, PPV Sequencing, and In Silico Assays

Macroscopic (virus-like) symptoms, i.e., leaf chlorotic rings and chlorotic fathering, were observed only in five plums, while no other symptoms were detected (Figure S1). Diagnostic analyses showed only the presence of PPV and PBNSPaV (Ct value: 15–19 and 21–31, respectively; Table S1). Eight plants (33%) showed a mixed PPV + PBNSPaV infection, while six (25%) and five (21%) plants were infected by PBNSPaV and PPV alone, respectively. The remaining five plants (21%) tested negative for the viruses investigated (and so were used as controls). PPV molecular typing revealed only the presence of the PPV-M strain. Four symptomatic plants tested positive for both PPV and PBNSPaV (i.e., PPV + PBNSPaV co-infection), while the remaining symptomatic one tested positive for PPV alone. No fruit production occurred in plants testing positive for PPV and/or PBNSPaV. 

### 2.2. Physiochemical Responses to Virus Infections

Table 1 shows the effects of PPV, PBNSPaV, and PPV + PBNSPaV infections on the investigated physiological and biochemical parameters. 

**Figure 1:**
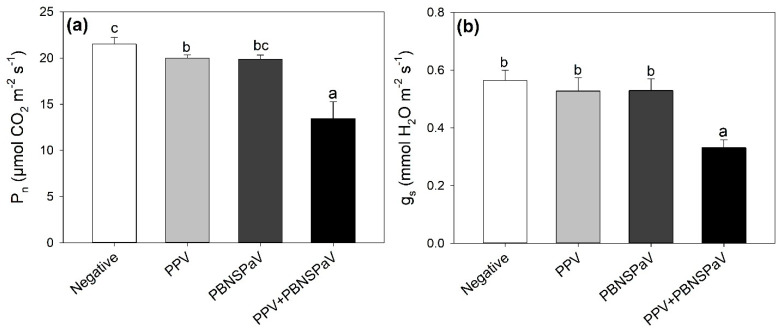
(**a**) Net photosynthesis (P_n_) and (**b**) stomatal conductance (g_s_) in plum cv. Coscia di Monaca leaves that tested negative for virus diagnosis (negative, white) and positive for plum pox virus (PPV, light gray), plum bark necrosis steam pitting associated virus (PBNSPaV, dark gray), and both viruses (PPV + PBNSPaV, black). Data are shown as mean ± standard deviation. Since two-way analysis of variance revealed a significant PPV × PBNSPaV interaction effect on both parameters, different letters indicate significant differences among means, according to Tukey’s post hoc test (*p* ≤ 0.05).

Net photosynthesis lightly decreased under PPV infection (−8%, in comparison with leaves that tested negative), and this detrimental effect was enhanced under PPV + PBNSPaV co-infection, reaching a −40% decrease (Figure 1a). Accordingly, g_s_ severely decreased (−40%) only under PPV + PBNSPaV co-infection (Figure 1b). Singular infection by PBNSPaV did not affect these photosynthetic parameters (Figure 1), and no significant changes due to any kind of virus infection were reported in C_i_ (Table 1).

Although no significant PPV + PBNSPaV interactive effects were reported on SPAD (Table 1), singular PPV and PBNSPaV infections showed an overall reduction in this parameter (PPV: 40 ± 3 vs. 35 ± 3, −12%; PBNSPaV: 39 ± 3 vs. 35 ± 3, −9%; data are shown as mean ± standard deviation). A similar response was observed in terms of Ψ_π_, but PPV alone induced a reduction in this parameter (2.41 ± 0.09 vs. 2.23 ± 0.08 −MPa, −7%), while PBNSPaV increased Ψ_π_ (2.26 ± 0.11 vs. 2.38 ± 0.06 −MPa, +5%). No significant effects were reported on Ψ_w_ and RWC (Table 1).

**Figure 2:**
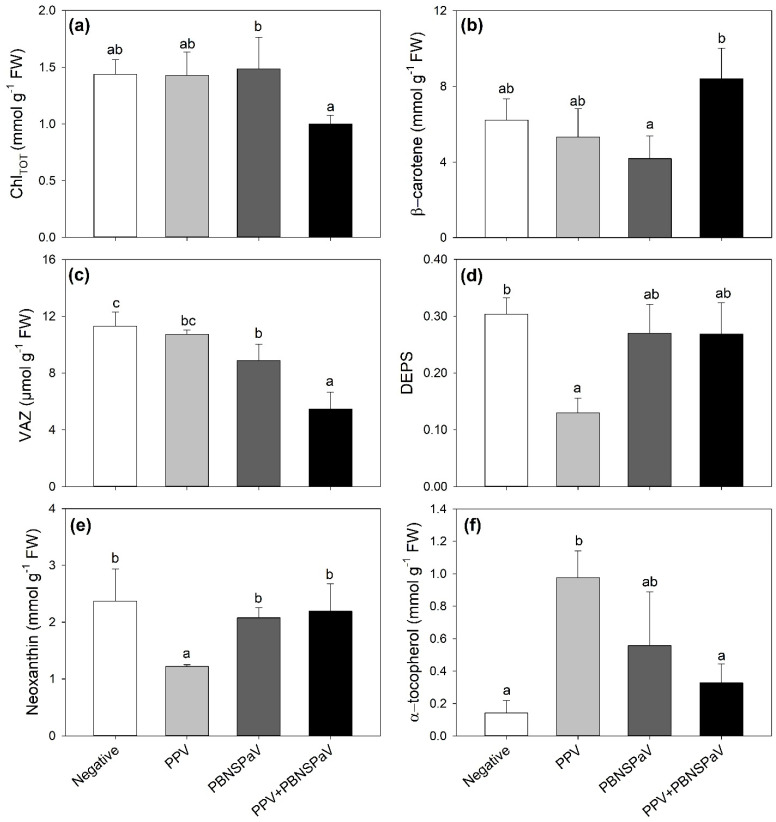
(**a**) Total chlorophyll (Chl_TOT_), (**b**) β-carotene, (**c**) violaxanthin + anteraxanthin + zeaxanthin (VAZ), (**d**) de-epoxidation state [DEPS, (anteraxanthin + zeaxanthin)/VAZ], (**e**) neoxanthin, and (**f**) α-tocopherol in plum cv. Coscia di Monaca leaves testing negative for virus diagnosis (negative, white) and positive for plum pox virus (PPV, light gray), plum bark necrosis steam pitting associated virus (PBNSPaV, dark gray), and both viruses (PPV + PBNSPaV, black). Data are shown as mean ± standard deviation. Since two-way analysis of variance revealed a significant PPV × PBNSPaV interaction effect on both parameters, different letters indicate significant differences among means, according to Tukey’s post hoc test (*p* ≤ 0.05). FW: fresh weight.

Only PPV + PBNSPaV co-infection significantly affected the Chl_TOT_ and β-carotene contents, with Chl_TOT_ decreasing by −25% and β-carotene showing a +19% higher content than under singular PBNSPaV infection (Figure 2a,b, respectively). VAZ decreased under PBNSPaV infection (−22%), and even more under PPV + PBNSPaV co-infection (−52%, Figure 2c), whereas DEPS and neoxanthin decreased only in leaves that tested positive for PPV alone (−58 and −49%, respectively; Figure 2d,e). Conversely, α-tocopherol severely increased only under PPV infection alone (−582%, Figure 2f). Although no significant PPV + PBNSPaV interactive effects were reported on lutein and β-, γ-, and δ- tocopherols (Table 1), the lutein content decreased in samples that tested positive for PPV [316 ± 12 vs. 247 ± 51 mmol g^−1^ fresh weight (FW), −21%] or to PBNSPaV (320 ± 31 vs. 243 ± 52 mmol g^−1^ FW, −24%), whereas γ-tocopherol increased only due to PPV (498 ± 104 vs. 708 ± 120 mmol g^−1^ FW, +42%) and β- and δ- tocopherols increased and decreased, respectively, only in PBNSPaV (β- tocopherol: 1444 ± 169 vs. 1790 ± 340 mmol g^−1^ FW, +24%; δ- tocopherol: 372 ± 114 vs. 215 ± 52 mmol g^−1^ FW, −42%).

**Figure 3:**
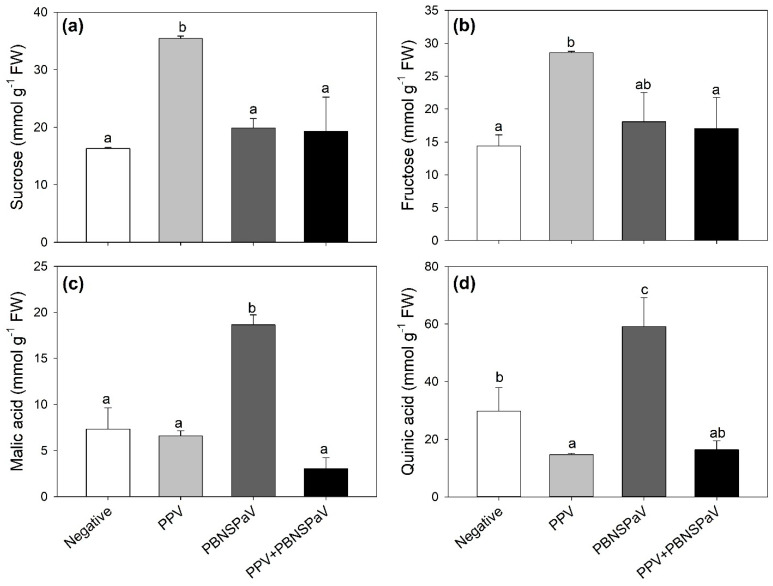
(**a**) Sucrose, (**b**) fructose, (**c**) malic acid, and (**d**) quinic acid contents in plum cv. Coscia di Monaca leaves testing negative for virus diagnosis (negative, white) and positive for plum pox virus (PPV, light gray), plum bark necrosis steam pitting associated virus (PBNSPaV, dark gray), and both viruses (PPV + PBNSPaV, black). Data are shown as mean ± standard deviation. Since two-way analysis of variance revealed a significant PPV × PBNSPaV interaction effect on both parameters, different letters indicate significant differences among means, according to Tukey’s post hoc test (*p* ≤ 0.05). FW: fresh weight.

Leaves testing positive only for PPV increased their sucrose and fructose contents (+117 and +99%, respectively; Figure 3a,b), whereas those infected only by PBNSPaV increased their malic and quinic acid contents (more than two-fold and +99%, respectively; Figure 3c,d), with quinic acid also showing a decrease under PPV infection alone (−51%). These parameters did not significantly change when the viruses occurred as co-infection (Figure 3). Furthermore, an overall PPV effect was reported on succinic acid [376 ± 63 vs. 257 ± 12 mmol g^−1^ FW, −32%], whereas an overall PBNSPaV effect occurred on glucose (66 ± 6 vs. 47 ± 9 mmol g^−1^ FW, +5%). No significant effects were reported in terms of citric acid (Table 1).

## 3. Discussion

The present study first confirmed that, despite the regulation that, since 2000, obliged farmers to destroy orchards when they were infected with PPV, PPV is still present in Tuscany (central Italy) [26], as in other Mediterranean and European areas [11,12,20,27], supporting the recent downregulation to RNQP [10]. The typical PPV-like symptoms, i.e., chlorotic fathering and chlorotic rings on leaves, observed in five plants were indeed confirmed using PCR assays. Molecular typing of PPV-infected samples generated NIb/CP amplicons corresponding to PPV-M, also confirming the overall higher occurrence of this strain, likely because PPV-M is characterized by an efficient transmission in fields by aphids [16]. Here, it is important to stress that although in 2019 the European Union regraded PPV to RNQP due to its widespread endemic presence, this virus still represents one of the most devastating pathogens of stone fruits, causing huge yield and economic losses worldwide. Thus, maintenance and the improvement of diagnostic and molecular characterization activities is still crucial, especially considering that the viruses and their vectors seem to be strongly favoured by climate change [28]. Interestingly, the present study also represents the first report of PBNSPaV in a Tuscany orchard. Indeed, although this virus has been largely reported in many areas of the world, including Africa, Asia, Europe, the Middle East, and North and South America, as well as in many different *Prunus* species [18,19,29,30,31,32], it was only recently reported once in a Japanese plum intercepted in a Tuscan nursery [20]. Conversely, despite their widespread field presence [11,33], MLRSV, PDV, PNRSV, ACLSV, and ApMV were here never detected in the collected samples. It is not surprising that all the five plants showing symptomatic leaves tested positive for PPV (although four as co-infection), as the major PBNSPaV symptoms are bark necrosis and steam pitting, the production of dark-coloured gum balls on scaffold branches, and necrosis on the woody cylinder (although variable leaf symptoms have been reported in susceptible species), not to mention that latent infections have been reported in several cultivars [34]. Most importantly, the presence of PPV and PBNSPaV occurring as both singular and co-infections opened up a great opportunity for the present study, which aimed to elucidate the physiological and biochemical responses of plum trees challenged by these viruses under real field conditions.

Compared to the large number of diagnostic and molecular characterization studies of plant viruses, fewer investigations have been carried out on the effects of these pathogens on plant physiology and metabolism. However, previous studies have shown that PPV infection produced ultrastructure alterations in *Prunus* cells, affecting the thylakoids, plastoglobolus, granal, and starch content and indicating that the chloroplast is the most affected cell organelle [13,22,23,35]. These changes were associated with severe detrimental effects of PPV on photosynthesis and carbohydrate metabolism, affecting photosystem II (PSII), directly or indirectly, by decreasing the amount of rubisco, oxygen-evolving enhancer, and PSII stability factors [36]. The harmful PPV effects were due to oxidative stress manifested as lipid peroxidation, protein oxidation, electrolyte leakage, the accumulation of hydrogen peroxide, and an imbalance in the antioxidant systems [35,37,38]. However, it should be noted that some conflicting results on the physiological alterations and damage to *Prunus* caused by PPV infection have been reported, likely due to a combination of variables, including metabolic differences among the plant species studied and differences in the process of infection, the level of PPV infection, the time of sampling, and the conditions under which the plants were grown [23,39]. Importantly, all previous investigations were carried out under controlled conditions. The present study not only confirmed, under real field conditions, the detrimental effects of PPV on the CO_2_ assimilation rate (decreased P_n_) due to both stomatal limitations (decreased g_s_) and mesophyll impairments (unchanged C_i_), but also showed that although PBNSPaV did not induce such photosynthetic impairments when occurring as a singular infection (although the leaf greenness was negatively affected by both viruses), it enhanced this photosynthetic damage when present as a co-infection with PPV. These outcomes add further complexity to the study of *Prunus*–PPV interaction, highlighting that it is also dependent on PPV presence as a singular or co-infection. Such infection-specific physiological responses were further highlighted by the outcomes of the leaf pigment and primary metabolite analyses (i.e., biochemical responses). 

The quantification of leaf pigments involved in light absorption is a clue to the actual functional capacity of photosystems [40]. Pigment changes due to PPV infection were previously reported (e.g., [41]), but never deeply investigated. Here, harsher effects on photosynthesis reported under PPV + PBNSPaV were confirmed by the severe reduction in the Chl_TOT_ content observed only under co-infection. This relationship between photosynthetic impairments and chlorophyll reduction was indeed largely reported under a number of biotic and abiotic stressors (e.g., [42,43]). Furthermore, the present study showed infection-specific responses in terms of both carotenoid and xanthophyll contents, i.e., accessory pigments used by plants in photosynthesis to enhance light harvesting in the blue–green spectral region. Specifically, the accumulation of β-carotene occurred only under PPV + PBNSPaV co-infection, suggesting a slight ability of the plant to use this compound (other than as accessory pigment) for its photoprotective properties through the quenching of both triplet chlorophyll (^3^Chl^*^) and singlet oxygen (^1^O_2_), i.e., the main reactive oxygen species (ROS) produced in photosynthetic organisms under stress conditions [44]. Accordingly, the xanthophyll cycle (VAZ), i.e., another photoprotective response activated by plants against oxidative stress [45], was also severely decreased under PPV + PBNSPaV co-infection, although a reduction in this parameter was also reported under singular PBNSPaV infection, suggesting that this virus can also affect the leaf biochemistry of *Prunus*. In fact, lutein, i.e., the most abundant xanthophyll in the photosynthetic apparatus of higher plants, with a specific property of quenching harmful ^3^Chl^*^, preventing ROS formation [46], was decreased due to both PPV and PBSNPaV (no significant PPV × PBSNPaV interaction was observed). However, PPV affected the pigment configuration the most, as the DEPS index decreased only under singular PPV infection, similarly to neoxanthin, which is a xanthophyll not directly involved in the xanthophyll cycle but for which has been reported a photoprotective role [47], whereas α-tocopherol increased. The rise in α-tocopherol, which is the major vitamin E compound found in leaf chloroplasts, where it is located in the chloroplast envelope, thylakoid membranes, and plastoglobuli, may have played a key role in abating the abovementioned photosynthetic impairments. Indeed, this antioxidant deactivates photosynthesis-derived ROS and prevents the propagation of lipid peroxidation by scavenging lipid peroxyl radicals in thylakoid membranes [48]. Other isoforms of tocopherols seemed to play a role in the *Prunus*–virus interaction: γ-tocopherol for PPV and β- and δ- tocopherols for PBNSPaV (no significant PPV × PBSNPaV interaction was observed).

Although some studies have shown that in the case of fruit tissues, PPV infection can modify the ripening process via an alteration of the primary metabolism, including sugars and organic acids (as well as secondary metabolism, including phenolic compounds [23]), no studies have investigated these compounds in leaf tissues. According to the abovementioned outcomes, the present study showed that infection-specific responses of primary metabolites also occurred in the leaves. Among sugars, sucrose and fructose increased only under singular PPV infection, although a PBNSPaV effect was reported on glucose. Llave [49] previously revealed that virus-impaired carbohydrate metabolism led to an increase in the free sugar content in leaves due to their use as a nitrogen and carbon source for replication and multiplication. Conversely, among organic acids, malic and quinic acids increased only under singular PBNSPaV infection, although a PPV effect was reported on succinic acid. These changes were canceled when PPV and PBNSPaV occurred in co-infection. Interestingly, these infection-specific changes seemed to result in different osmotic regulations, as Ψ_π_ showed different changes between PPV (increased and decreased, respectively). These osmotic regulations were not twinned with changes in Ψ_w_ and RWC, suggesting that both viruses, occurring both individually and as a co-infection, did not affect the water status of plants, as previously reported in kale leaves infected by turnip mosaic virus [50]. Further investigation of these parameters (and many others) would also be interesting in fruit tissue, but unfortunately no fruit production was reported in plants testing positive to PPV and/or PBNSPaV.

In conclusion, although the autochthonous plum varieties were evaluated from a chemical and nutraceutical point of view, the present study represents the first virogical investigation of this plant material. Specifically, (1) among the several investigated viruses, plums tested positive only for PPV and PBNSPaV (first reported in a Tuscany orchard), occurring as both singular and co-infections; (2) the detrimental effects of PPV on photosynthetic performance were confirmed, and although PBNSPaV did not induce such impairments when occurring as a singular infection, it enhanced this damaging effect when present as a co-infection with PPV; and (3) infection-specific responses in terms of accessory pigments (i.e., carotenoids and xanthophylls), as well as sugars and organic acids, occurred, this being likely related to photoprotective mechanisms and osmotic regulations activated by plums under virus-induced oxidative stress. Overall, the results here presented represent an important step to fill knowledge gaps about the interaction of plant viruses and autochthonous *Prunus* cultivars, and we believe it may encourage other similar research to achieve more accurate data on virus populations at both national and international levels. More research should be carried out to improve our knowledge of plum virus diffusion and PPV and PBNSPaV genetic features, as well as of the effects of these regulated non-quarantine pests on plum trees, also investigating their fruits, representing an important source of food and health.

## 4. Materials and Methods

### 4.1. Plant Material and Experimental Design

In 2022, experimental activities were carried out in a three-year-old *P. domestica* cv. Coscia di Monaca (autochthonous) orchard located in Arezzo, Tuscany (central Italy, 43°27′47″ N, 11°52′41″ E, 296 m a.s.l.). The orchard was organized into rows with a north–south orientation and had an anti-insect and anti-hail net covering the plants, a turf covering under the rows, and a drip irrigation system to keep plants well-watered. The experimental area is characterized by a hot-summer Mediterranean climate (Csa, Köppen, and Geiger classification; [51]), with annual mean, minimum, and maximum temperatures of 13.0, 3.6 (January), and 23.2 °C (July), respectively, and an annual rainfall of around 1014 mm. The soil type is loam (26% clay, 49% silt, and 25% sand). The average soil pH, organic matter, and phosphorous and potassium contents were 7, 2%, 14 mg kg^−1^, and 780 mg kg^−1^, respectively. 

In June, 24 plants were selected for size uniformity, and one leaf per plant, which was randomly selected among the youngest mature (located in the middle–upper part of the canopy) and sun-exposed ones, was first measured in the field (from 10.00 am to 13.00 pm, under clear sky conditions) for physiological characterization, and then sampled for further diagnostic and biochemical analyses. All collected samples were kept refrigerated until quickly reaching the Plant Pathology Lab at the Department of Agriculture, Food and Environment of the University of Pisa. Here, the leaf samples for molecular diagnosis were immediately handled for total nucleic acid (TNA) extraction, whereas the leaf samples for biochemical analyses were stored at −80 °C.

### 4.2. RNA Isolation and cDNA Synthesis

TNA was recovered from 500 mg of leaf tissue with cetyltrimethylammonium (CTAB) buffer, according to Li et al. [52] with some modifications [53]. Briefly, the leaves were powdered in liquid nitrogen with 5 mL CTAB 2% (*w*/*v*) buffer. After incubation at 65 °C for 15 min, one volume of chloroform:iso-amyl alcohol (24:1, *v*/*v*) was added and TNA was precipitated with one volume of isopropanol. The pellet was then washed with 70% ethanol (*v*/*v*), air-dried, and dissolved in 80 μL RNase/DNase-free water. An RNA purification kit (EURx, Gdańsk, Poland) was used, and the samples were stored at −80 °C. The synthesis of cDNA was finally performed using M-MMLV reverse transcriptase (GeneSpin S.R.L., Milan, Italy), according to the manufacturer’s instructions, and kept at −20 °C until it was utilized.

### 4.3. Virus Detection

The PCR amplification was performed in a Rotor Gene Q thermocycler (Qiagen, Venlo, The Netherlands) using quantitative PCR protocols targeting coat protein gene (CP) for PPV [54], ACLSV [55], PDV, and PNRSV [56] and heat shock protein gene (hHSP70) for PBNSPaV [57]. Moreover, end-point PCR was conducted in a C1000 Touch thermocycler (Bio-Rad, Hercules, CA, USA) for ApMV using a protocol spanning CP [58]. The amplified products were analysed with electrophoresis in a 2% agarose gel and the DNA bands visualized using UV lights. Infected, healthy, and no-template controls were included in each assay.

Myrobalan latent ringspot virus (MLRSV) presence was instead assayed using a kit based on a double antibody sandwich enzyme-linked immunosorbent assay (DAS-ELISA) test, following the manufacturer’s instructions (Agritest, Valenzano, Italy).

### 4.4. PPV Strain Characterization

Molecular typing of the PPV strains (i.e., PPV-D, PPV-M, and PPV-Rec) was carried out in a C1000 Touch thermocycler (Bio-Rad Laboratories) using primers spanning nuclear inclusion bodies and the coat protein (NIb/CP) region [59]. Infected, healthy, and no-template controls were included as references.

### 4.5. Leaf Gas Exchange and Greenness

The net photosynthesis (P_n_), stomatal conductance (g_s_), and intercellular CO_2_ concentration (C_i_) were determined using a Li-6800 portable photosynthesis system equipped with a 6800-01A LED light source (Li-Cor, Lincoln, NE, USA) operating at 410 ppm CO_2_ concentration and saturating light conditions (1700 µmol m^−2^ s^−1^ photosynthetically active radiation). 

A SPAD 502 (Konika Minolta, Chiyoda, Tokyo, Japan) was used to determine the leaf greenness (SPAD). Three measurements per leaf were collected, and the mean of these measurements was recorded.

### 4.6. Leaf Water Status

Water status parameters were determined on the same or closest leaves used for gas exchange and Chl_SPAD_ at mid-day, according to Cotrozzi et al. [60]. Ψ_w_ was measured on a leaf cut with a sharp razor blade, inserted into a rubber stopper, and then placed in a Scholander-type pressure chamber (model 600; PMS Instrument, Albany, OR, USA). To determine Ψ_π_, a leaf portion was placed in a mesh insert into a microcentrifuge tube, immersed in liquid nitrogen, and then stored at −20 °C until processing. The osmolality was determined with a Wescor 5500 vapour pressure osmometer (Wescor, Logan, UT, USA), and Ψ_π_ was converted from osmolality using the Van’t Hoff equation. Another leaf portion was used to determine the relative water content (RWC), which was calculated as (FW − DW)/(TW − DW) × 100, where FW is the fresh weight, TW is the turgid weight after rehydrating the samples for 24 h, and DW is the dry weight after oven-drying the leaves at 60 °C until a constant weight.

### 4.7. Leaf Pigment Contents

Leaf pigments were determined with ultra-high performance liquid chromatography (UHPLC) using a Dionex UltiMate 3000 system equipped with an Acclaim 120 C18 column (5 μm particle size, 4.6 mm internal diameter × 150 mm length), maintained in a Dionex TCC-100 column oven at 30 °C, and a Dionex UVD 170U detector (Thermo Scientific, Waltham, MA, USA; [61]). The leaf material (50 mg FW) was homogenized in 1 mL of 100% HPLC-grade methanol and incubated overnight at 4 °C in the dark. The sample supernatants were filtered through 0.2 μm Minisart^®^ SRT 15 aseptic filters. The pigments were eluted using 100% solvent A (acetonitrile/methanol, 75/25, *v*/*v*) for the first 14 min to elute xanthophylls (neoxanthin, Neo; violaxanthin, Vio; antheraxanthin, Ant; lutein, Lut; zeaxanthin, Zea; in order of elution), followed by a 1.5 min linear gradient to 100% solvent B (methanol/ethylacetate, 68/32, *v*/*v*), which was pumped for 14.5 min to elute chlorophyll *b* (Chl *b*) and *a* (Chl *a*) and β-carotene (β-car), followed by a 2 min linear gradient to 100% solvent A. The flow rate was 1 mL min^−1^. The column was allowed to re-equilibrate in 100% solvent A for 1 min before the next injection. Chlorophylls, xanthophylls, and carotenes were detected using their absorbance at 445 nm, and tocopherols (α, β, γ, δ—isomers) at 295 nm. To quantify the pigment content, known amounts (0.003–0.5 mg mL^−1^) of pure standards (Sigma-Aldrich, St. Louis, MO, USA) were injected into the UHPLC system and an equation correlating the peak area to the pigment concentration was formulated. The chromatographic data were processed and recorded using Chromeleon Chromatography Management System software, version 7.2.10–2019 (Thermo Scientific). The sum of all compounds from the specific group identified in the study was calculated as follows: the total chlorophyll content (Chl_TOT_) was calculated as Chl *a* + Chl *b*, the total carotenoid content (Car_TOT_) was calculated as Neo + Vio + Ant + Lut + Zea + β-car, while the xanthophyll cycle pigment content (VAZ) was calculated as Vaz + Ant + Zea. The de-epoxidation state (DEPS) was calculated as (Ant + Zea)/VAZ.

### 4.8. Leaf Sugar and Organic Acid Contents

Soluble sugars (i.e., D-glucose, D-fructose, and sucrose) were measured using the K-SUFRG commercial kit (Megazyme, Wicklow, Ireland) following the manufacturer’s protocol. After extraction with ethanol 80% (*v*/*v*), D-glucose, D-fructose, and sucrose were determined with a spectrophotometer (UV-1900 UV-vis, Shimadzu, Kyoto, Japan) at 340 nm. Organic acids (i.e., citric, malic, shikimic, and quinic acids) were determined according to Eyéghé-Bickong et al. [62], with minor modifications. After extraction with 100% HPLC-demineralized water, these organic acids were measured with the same UHPLC reported above equipped with a pre-column Repromer H (8 mm internal diameter × 20 mm length, 9 μm particle size) and a Repromer H column (8 mm internal diameter × 300 mm length, 9 μm particle size) using 9 mM sulphuric acid as the eluent and a flow rate of 1 mL min^−1^. Organic acids were detected using their absorbance at 210 nm with the same detector reported above. To quantify their content, known amounts (0.003–0.5 mg ml^−1^) of pure standards (Sigma-Aldrich, St. Louis, MO, USA) were injected into the UHPLC system and an equation correlating the peak area to the organic acid concentration was formulated.

### 4.9. Statistical Analysis

The Shapiro–Wilk test was used to evaluate the normal distribution of the leaf physiological and biochemical parameters. The effects of PPV, PBNSPaV, and their interaction PPV/PBNSPaV (i.e., mixed infection) on the investigated parameters were assessed with a two-way analysis of variance (ANOVA), followed by Tukey’s post hoc test. The statistical analysis was performed in JMP 13.0 (SAS Institute, Cary, NC, USA), and significant differences were considered for *p* ≤ 0.05.

## Figures and Tables

**Table 1:** F values and *p* levels (***: *p* ≤ 0.001, **: *p* ≤ 0.01, *: *p* ≤ 0.05, ns: *p* > 0.05) of two-way analysis of variance for the effects of plum pox virus (PPV), plum bark necrosis steam pitting associated virus (PBNSPaV), and PPV + PBNSPaV infections on leaf physiological and biochemical parameters of plum cv. Coscia di Monaca. Parameter abbreviations: P_n_, net photosynthesis; g_s_, stomatal conductance; C_i_, intercellular CO_2_ concentration; SPAD, leaf greenness estimated as SPAD index; Ψ_w_, water potential; Ψ_π_, osmotic potential; RWC, relative water content; VAZ, violaxanthin + antheraxanthin + zeaxhanthin content; DEPS, de-epoxidation state [(anteraxanthin + zeaxanthin)/VAZ].

Parameters	PPV	PBNSPaV	PPV + PBNSPaV
P_n_	643.61 ***	616.77 ***	234.71 ***
g_s_	235.16 ***	242.30 ***	112.46 **
C_i_	0.10 ns	0.40 ns	34.19 ns
SPAD	279.30 ***	162.30 **	0.04 ns
Ψ_w_	0.22 ns	0.51 ns	0.021 ns
Ψ_π_	145.04 **	70.41 *	0.46 ns
RWC	44.88 ns	0.01 ns	23.94 ns
Chl_TOT_	31.22 ns	224.16 ***	36.42 ***
β-carotene	28.78 ns	0.01 ns	89.31 *
VAZ	162.12 **	609.34 ***	83.41 *
DEPS	49.93 *	18.00 ns	47.97 *
Lutein	99.17 **	122.73 **	10.82 ns
Neoxanthin	73.65 *	31.24 ns	110.12 **
α-tocopherol	92.11 *	13.75 ns	285.14 ***
β-tocopherol	45.64 ns	60.46 *	24.67 ns
γ-tocopherol	137.40 **	42.79 ns	0.24 ns
δ-tocopherol	13.66 ns	75.55 *	0.11 ns
Glucose	13.97 ns	5.19 *	37.01 ns
Fructose	79.51 *	20.95 ns	59.31 *
Sucrose	359.46 ***	165.90 ***	407.37 ***
Citric acid	0.13 ns	0.01 ns	0.17 ns
Malic acid	506.13 ***	114.37 **	42.05 ***
Succinic acid	68.30 *	42.73 ns	31.95 ns
Quinic acid	752.21 ***	218.44 ***	172.05 **

## Data Availability

Data sharing not applicable.

## Supplementary Materials

The following supporting information can be downloaded at: https://www.mdpi.com/article/10.3390/plants12183264/s1, Figure S1: Leaf of plum cv. Coscia di Monaca infected by (**A**) plum pox virus (PPV) and plum bark necrosis steam pitting associated virus (PBNSPaV), (**B**) only PPV, (**C**) only PBNSPaV, and (**D**) tested negative to both PPV and PBNSPaV (i.e., healthy). Leaf chlorotic rings and chlorotic fathering symptoms were observed only in leaves of plants infected by plum pox virus; Table S1: Ct values from diagnosis assessment carried out on the 24 plum trees cv. Coscia di Monaca selected in Arezzo district (Tuscany, Central Italy). Presence of apple chlorotic leaf spot virus (ACLSV), apple mosaic virus (ApMV), myrabolan latent ringpot virus (MLRSV), plum bark necrosis steam pitting associated virus (PBNSPaV), prune dwarf virus (PDV), prunus necrotic ringspot virus (PNRSV), and plum pox virus (PPV) were assayed.

## References

[1] Jo Y., Choi H., Lian S., Cho J.K., Chu H., Cho W.K. (2020). Identification of Viruses Infecting Six Plum Cultivars in Korea by RNA-Sequencing. PeerJ.

[2] Manco R., Basile B., Capuozzo C., Scognamiglio P., Forlani M., Rao R., Corrado G. (2019). Molecular and Phenotypic Diversity of Traditional European Plum (*Prunus domestica* L.) Germplasm of Southern Italy. Sustainability.

[3] Sottile F., Impallari F.M., Peano C., Giuggioli N.R. (2010). Antioxidant Compounds and Qualitative Traits in European (*Prunus domestica* L.) and Japanese (*P. triflora* L.) Plum Fruits as Affected by Cold Storage. Acta Hortic..

[4] Food and Agriculture Organization of the United Nations (2022). Food and Agriculture Data. https://www.fao.org/faostat/en/#home.

[5] Ionica M.E., Nour V., Trandafir I., Cosmulescu S., Botu M. (2013). Physical and Chemical Properties of Some European Plum Cultivars (*Prunus domestica* L.). Not. Bot. Horti Agrobot. Cluj.

[6] Žežlina I., Rot M., Kač M., Trdan S. (2016). Causal Agents of Stone Fruit Diseases in Slovenia and the Potential for Diminishing Their Economic Impact—A Review. Plant Prot. Sci..

[7] Rizza S., Conti F., Pasquini G., Tessitori M. (2014). First Report of Plum Pox Virus Strain M Isolates in Apricot in Sicily, Italy. Plant Dis..

[8] Umer M., Liu J., You H., Xu C., Dong K., Luo N., Kong L., Li X., Hong N., Wang G. (2019). Genomic, Morphological and Biological Traits of the Viruses Infecting Major Fruit Trees. Viruses.

[9] Rubio M., Martínez-Gómez P., Marais A., Sánchez-Navarro J.A., Pallás V., Candresse T. (2017). Recent Advances and Prospects in Prunus Virology: Prunus Virology. Ann. Appl. Biol..

[10] European Union (2019). Regulations-Commission Implementing Regulation (EU) 2019/2072 of 28 November 2019 Establishing Uniform Conditions for the Implementation of Regulation (EU) 2016/2031 of the European Parliament and the Council, as Regards Protective Measures against Pests of Plants, and Repealing Commission Regulation (EC) No 690/2008 and Amending Commission Implementing Regulation (EU) 2018/2019. https://eur-lex.europa.eu/legal-content/EN/TXT/?uri=celex%3A32019R2072.

[11] Gospodaryk A., Moročko-Bičevska I., Pūpola N., Kāle A. (2013). Occurrence of Stone Fruit Viruses in Plum Orchards in Latvia. Proceedings of the Latvian Academy of Sciences. Section B. Nat. Exact Appl. Sci..

[12] Zagrai L.A., Zagrai I., Guzu G.M., Roșu-Mareș S.D., Moldovan C. (2022). Assessment of the Virus Infections Occurrence in New Established Plum and Sweet Cherry Orchards in Transylvania, Romania. Not. Bot. Horti Agrobot..

[13] Kegler H., Hartmann W., Hadidi A., Khetarpal R.K., Koganezawa H. (1998). Present Status of Controlling Conventional Strains of Plum Pox Virus. Plant Virus Disease Control.

[14] Cambra M., Capote N., Myrta A., Llácer G. (2006). Plum Pox Virus and the Estimated Costs Associated with Sharka Disease. EPPO Bull..

[15] De Mori G., Savazzini F., Geuna F., Poltronieri P., Hong Y. (2020). Molecular Tools to Investigate Sharka Disease in Prunus Species. Applied Plant Biotechnology for Improving Resistance to Biotic Stress.

[16] James D., Varga A., Sanderson D. (2013). Genetic Diversity of Plum Pox Virus: Strains, Disease and Related Challenges for Control. Can. J. Plant Pathol..

[17] Myrta A., Di Terlizzi B., Savino V., Martelli G.P. (2003). Virus Diseases Affecting the Mediterranean Stone Fruit Industry: A Decade of Surveys. Virus and Virus-Like Diseases of Stone Fruits, with Particular Reference to the Mediterranean Region. Options Méditerranéennes.

[18] Marini D.B., Zhang Y.-P., Rowhani A., Uyemoto J.K. (2002). Etiology and Host Range of a Closterovirus Associated with Plum Bark Necrosis-Stem Pitting Disease. Plant Dis..

[19] Candresse T., Faure C., Theil S., Marais A. (2017). First Report of Plum Bark Necrosis Stem Pitting-Associated Virus Infecting Flowering Cherry in Japan. Plant Dis..

[20] Drosera L., Gilli G., Ciardelli C. (2022). Rapporto Annuale Attività 2021 Servizio Fitosanitario Regionale.

[21] Fraile A., García-Arenal F. (2010). The Coevolution of Plants and Viruses. Advances in Virus Research.

[22] Clemente-Moreno M.J., Hernández J.A., Diaz-Vivancos P. (2015). Sharka: How Do Plants Respond to Plum Pox Virus Infection?. J. Exp. Bot..

[23] Espinoza C., Bascou B., Calvayrac C., Bertrand C. (2021). Deciphering Prunus Responses to PPV Infection: A Way toward the Use of Metabolomics Approach for the Diagnostic of Sharka Disease. Metabolites.

[24] Sottile F., Girgenti V., Giuggioli N.R., Del Signore M.B., Peano C. (2015). Quality of Autochthonous Sicilian Plums. Ital. J. Food Sci..

[25] Ceccarelli D., Antonucci F., Talento C., Ciccoritti R. (2021). Chemical Characterization in the Selection of Italian Autochthonous Genotypes of Plum. Sci. Hortic..

[26] Ginanni M., Materazzi A., Mainardi M., Triolo E. (1993). La “Vaiolatura” Delle Drupacee: Indagini Sulla Presenza in Toscana. Inf. Fitopatol..

[27] Yardimci B.C.N., Culal-Klllc H. (2011). Detection of Viruses Infecting Stone Fruits in Western Mediterranean Region of Turkey. Plant Pathol. J..

[28] IPPC Secretariat (2021). Scientific Review of the Impact of Climate Change on Plant Pests.

[29] Abou Ghanem-Sabanadzovic N., Mahboubi M., Di Terlizzi B., Sabanadzovic S., Savino V., Uyemoto J.K., Martelli G.P. (2001). Molecular Detection of A Closterovirus Associated with Apricot Stem Pitting in Southern Italy. J. Plant Pathol..

[30] Sánchez-Navarro J.A., Aparicio F., Herranz M.C., Minafra A., Myrta A., Pallás V. (2005). Simultaneous Detection and Identification of Eight Stone Fruit Viruses by One-Step RT-PCR. Eur. J. Plant Pathol..

[31] Zamorano A., Chiumenti M., Fernández C., Quiroga N., Pino A.M., Sagredo K., Saldarelli P., Fiore N. (2017). First Report of Cherry Virus A and Plum Bark Necrosis Stem Pitting-Associated Virus in Cherry in Chile. Plant Dis..

[32] Bester R., Maree H.J. (2020). First Report of Plum Bark Necrosis Stem Pitting-Associated Virus in Japanese Plums in South Africa. Plant Dis..

[33] Mahfoudhi N., El Air M., Moujahed R., Salleh W., Djelouah K. (2013). Occurrence and Distribution of Pome Fruit Viruses in Tunisia. Phytopathol. Mediterr..

[34] Marini D.B., Rowhani A., Uyemoto J.K. (2002). Graft-Transmissible Agent Causes Bark Necrosis and Stem Pitting in Plum Trees. Calif. Agric..

[35] Hernandez J.A., Rubio M., Olmos E., Ros-Barcelo A., Martinez-Gomez P. (2004). Oxidative Stress Induced by Long-Term Plum Pox Virus Infection in Peach (*Prunus persica*). Physiol. Plant.

[36] Clemente-Moreno M.J., Díaz-Vivancos P., Rubio M., Fernández-García N., Hernández J.A. (2013). Chloroplast Protection in Plum Pox Virus-Infected Peach Plants by L-2-Oxo-4-Thiazolidine-Carboxylic Acid Treatments: Effect in the Proteome: Chloroplast Protection by OTC. Plant Cell Environ..

[37] Diaz-Vivancos P., Clemente-Moreno M.J., Rubio M., Olmos E., Garcia J.A., Martinez-Gomez P., Hernandez J.A. (2008). Alteration in the Chloroplastic Metabolism Leads to ROS Accumulation in Pea Plants in Response to Plum Pox Virus. J. Exp. Bot..

[38] Hernandez J.A., Diaz-Vivancos P., Rubio M., Olmos E., Ros-Barcelo A., Martinez-Gomez P. (2006). Long-Term Plum Pox Virus Infection Produces an Oxidative Stress in a Susceptible Apricot, *Prunus Armeniaca*, Cultivar but Not in a Resistant Cultivar. Physiol. Plant.

[39] Diaz-Vivancos P., Rubio M., Mesonero V., Periago P., Ros Barcelo A., Martinez-Gomez P., Hernandez J. (2006). The Apoplastic Antioxidant System in *Prunus*: Response to Long-Term Plum Pox Virus Infection. J. Exp. Bot..

[40] Pinto-Marijuan M., Munne-Bosch S. (2014). Photo-Oxidative Stress Markers as a Measure of Abiotic Stress-Induced Leaf Senescence: Advantages and Limitations. J. Exp. Bot..

[41] Baumgartnerová H., Slováková L., Petrusová N. (1998). Relationship between Concentration of Plum Pox Virus and Content of Pigments in Virus-Infected Symptomatic Apricot Leaves. Acta Virol..

[42] Cotrozzi L., Remorini D., Pellegrini E., Guidi L., Nali C., Lorenzini G., Massai R., Landi M. (2018). Living in a Mediterranean City in 2050: Broadleaf or Evergreen ‘Citizens’?. Environ. Sci. Pollut. Res..

[43] Pellegrini E., Cotrozzi L., Neri L., Baraldi R., Carrari E., Nali C., Lorenzini G., Paoletti E., Hoshika Y. (2021). Stress Markers and Physiochemical Responses of the Mediterranean Shrub *Phillyrea angustifolia* under Current and Future Drought and Ozone Scenarios. Environ. Res..

[44] Havaux M. (2014). Carotenoid Oxidation Products as Stress Signals in Plants. Plant J..

[45] Latowski D., Kuczyńska P., Strzałka K. (2011). Xanthophyll Cycle—A Mechanism Protecting Plants against Oxidative Stress. Redox Rep..

[46] Jahns P., Holzwarth A.R. (2012). The Role of the Xanthophyll Cycle and of Lutein in Photoprotection of Photosystem II. BBA-Bioenerg..

[47] Giossi C., Cartaxana P., Cruz S. (2020). Photoprotective Role of Neoxanthin in Plants and Algae. Molecules.

[48] Munné-Bosch S. (2005). The Role of -Tocopherol in Plant Stress Tolerance. J. Plant Physiol..

[49] Llave C. (2016). Dynamic Cross-Talk between Host Primary Metabolism and Viruses during Infections in Plants. Curr. Opin. Virol..

[50] Şevïk M.A., Cansiz N. (2021). The Impact of Turnip Mosaic Virus on Physiological and Morphological Parameters of Kale Plants. Gümüşhane Üniversitesi Fen Bilim. Enstitüsü Derg..

[51] Beck H.E., Zimmermann N.E., McVicar T.R., Vergopolan N., Berg A., Wood E.F. (2018). Present and Future Köppen-Geiger Climate Classification Maps at 1-Km Resolution. Sci. Data.

[52] Li R., Mock R., Huang Q., Abad J., Hartung J., Kinard G. (2008). A Reliable and Inexpensive Method of Nucleic Acid Extraction for the PCR-Based Detection of Diverse Plant Pathogens. J. Virol. Methods.

[53] Pedrelli A., Panattoni A., Cotrozzi L. (2023). First Molecular Characterization of Plum Pox Virus Strains in Stone Fruits of Tuscany (Central Italy). J. Plant Pathol..

[54] Olmos A., Bertolini E., Gil M., Cambra M. (2005). Real-Time Assay for Quantitative Detection of Non-Persistently Transmitted Plum Pox Virus RNA Targets in Single Aphids. J. Virol. Methods.

[55] Osman F., Al Rwahnih M., Rowhani A. (2016). Real-Time RT-qPCR Detection of Cherry Rasp Leaf Virus, Cherry Green Ring Mottle Virus, Cherry Necrotic Rusty Mottle Virus, Cherry Virus A and Apple Chlorotic Leaf Spot Virus in Stone Fruits. J. Plant Pathol..

[56] Kim B.T., Gibson P.G., Scott S.W. (2010). Expression of the Coat Protein Genes of PNRSV and PDV in the Synergistic Disease Peach Stunt. Proceedings of the 21st International Conference on Virus and other Graft Transmissible Diseases of Fruit Crops.

[57] Al Rwahnih M., Uyemoto J.K., Falk B.W., Rowhani A. (2007). Molecular Characterization and Detection of Plum Bark Necrosis Stem Pitting-Associated Virus. Arch. Virol..

[58] Menzel W., Jelkmann W., Maiss E. (2002). Detection of Four Apple Viruses by Multiplex RT-PCR Assays with Coamplification of Plant MRNA as Internal Control. J. Virol. Methods.

[59] Subr Z., Pittnerová S., Glasa M. (2004). A Simplified RT-PCR-Based Detection of Recombinant Plum Pox Virus Isolates. Acta Virol..

[60] Cotrozzi L., Peron R., Tuinstra M.R., Mickelbart M.V., Couture J.J. (2020). Spectral Phenotyping of Physiological and Anatomical Leaf Traits Related with Maize Water Status. Plant Physiol..

[61] Zhang L., Hoshika Y., Carrari E., Cotrozzi L., Pellegrini E., Paoletti E. (2018). Effects of Nitrogen and Phosphorus Imbalance on Photosynthetic Traits of Poplar Oxford Clone under Ozone Pollution. J. Plant Res..

[62] Eyéghé-Bickong H.A., Alexandersson E.O., Gouws L.M., Young P.R., Vivier M.A. (2012). Optimisation of an HPLC Method for the Simultaneous Quantification of the Major Sugars and Organic Acids in Grapevine Berries. J. Chromatogr. B.

