# Supplementary material for: Physiological and Biochemical Responses Induced by Plum Pox Virus and Plum Bark Necrosis Steam Pitting Associated Virus in Tuscany Autochthonous Plum cv. Coscia di Monaca
Source: Plants (Basel). 2023 Sep 14;12(18):3264. doi: 10.3390/plants12183264 (PMC10535208; doi:10.3390/plants12183264)
Supplement: Supplementary file 1 [file plants-12-03264-s001.zip › plants-2570073-supplementary.pdf]

plants

MDPI

Supplementary Material

Physiological and biochemical responses induced by plum pox virus and plum bark necrosis steam pitting associated virus in Tuscany autochthonous plum cv. Coscia di Monaca

Athos Pedrelli, Gian Piero Ricci, Alessandra Panattoni, Cristina Nali *, and Lorenzo Cotrozzi

Department of Agriculture, Food and Environment, University of Pisa, Via del Borghetto 80, 56124 Pisa, Italy; athos.pedrelli@phd.unipi.it (A.P.); g.ricci31@studenti.unipi.it (G.P.R.); alessandra.panattoni@unipi.it (A.P.); lorenzo.cotrozzi@unipi.it (L.C.)

* Correspondence: cristina.nali@unipi.it; Tel.: +39-050-2210552

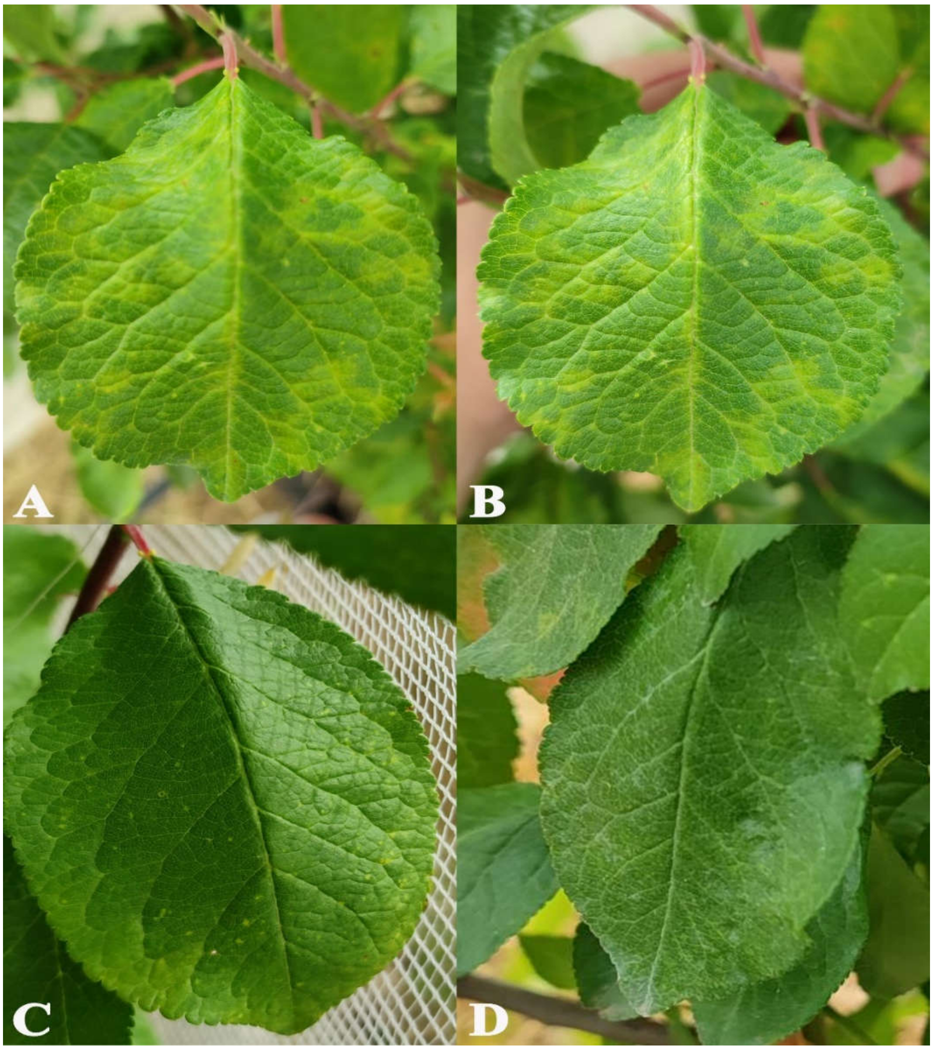

Figure S1. Leaf of plum cv. Coscia di Monaca infected by (**A**) plum pox virus (PPV) and plum bark necrosis steam pitting associated virus (PBNSPaV), (**B**) only PPV, (**C**) only PBNSPaV, and (**D**) tested negative to both PPV and PBNSPaV (i.e., healthy). Leaf chlorotic rings and chlorotic fathering symptoms were observed only in leaves of plants infected by plum pox virus.

plants

MDPI

Table S1. Ct values from diagnosis assessment carried out on the 24 plum trees cv. Coscia di Monaca selected in Arezzo district (Tuscany, Central Italy). Presence of apple chlorotic leaf spot virus (ACLSV), apple mosaic virus (ApMV), myrabolan latent ringpot virus (MLRSV), plum bark necrosis steam pitting associated virus (PBNSPaV), prune dwarf virus (PDV), prunus necrotic ringspot virus (PNRSV), and plum pox virus (PPV) were assayed.

Sample	ACLSV	ApMV	MLRSV	PBNSPaV	PDV	PNRSV	PPV
1	-	-	-	-	-	-	-
2	-	-	-	-	-	-	16.36
3	-	-	-	29.51	-	-	-
4	-	-	-	24.01	-	-	15.78
5	-	-	-	-	-	-	17.12
6	-	-	-	22.23	-	-	15.67
7	-	-	-	21.95	-	-	16.83
8	-	-	-	22.02	-	-	19.03
9	-	-	-	22.63	-	-	19.91
10	-	-	-	22.93	-	-	-
11	-	-	-	-	-	-	-
12	-	-	-	23.55	-	-	-
13	-	-	-	-	-	-	18.02
14	-	-	-	-	-	-	-
15	-	-	-	22.26	-	-	-
16	-	-	-	27.14	-	-	18.45
17	-	-	-	-	-	-	-
18	-	-	-	23.23	-	-	-
19	-	-	-	29.67	-	-	16.72
20	-	-	-	-	-	-	19.55
21	-	-	-	27.46	-	-	-
22	-	-	-	23.23	-	-	15.79
23	-	-	-	-	-	-	-
24	-	-	-	-	-	-	17.91
